# Heat-Induced Changes in the Physical Properties of a New Premixed Calcium Silicate-Containing Root Canal Sealer: An In Vitro Study

**DOI:** 10.3390/dj12040106

**Published:** 2024-04-12

**Authors:** Ryota Ito, Satoshi Watanabe, Akira Kouno, Shinya Yamauchi, Takashi Okiji

**Affiliations:** Department of Pulp Biology and Endodontics, Division of Oral Health Sciences, Graduate School of Medical and Dental Sciences, Tokyo Medical and Dental University (TMDU), 1-5-45 Yushima, Bunkyo-ku, Tokyo 113-8549, Japan; itohendo@tmd.ac.jp (R.I.); akirabird720@yahoo.co.jp (A.K.); yamauchishinnya@yahoo.co.jp (S.Y.); t.okiji.endo@tmd.ac.jp (T.O.)

**Keywords:** calcium silicate, heating, material testing, root canal filling materials

## Abstract

This study aimed to examine how heating affects the physical properties of a newly developed premixed calcium silicate-containing sealer (AH Plus Bioceramic Sealer; AHB), in comparison with EndoSequence BC Sealer (ES), AH Plus Jet (AH), and Pulp Canal Sealer. The setting time, flow, and film thickness were tested with or without heating at 100 °C for 30 or 60 s, in accordance with ISO6876:2012 standards. Ultrastructural and elemental analyses were performed with scanning electron microscopy (SEM) and energy-dispersive X-ray spectroscopy (EDS). Data were analyzed using one-way analysis of variance (ANOVA) with a Tukey post hoc test (α = 0.05). All sealers exhibited significantly shorter setting time and flow after heating at 100 °C for 30 and 60 s (*p* < 0.05). After heating, AHB showed a significantly higher film thickness compared to that of the other materials (*p* < 0.05). None of the tested properties of heat-applied AHB and ES met ISO standards, except the setting time in ES. The SEM/EDS results for AHB and ES were not affected by heating. The detected changes in physical properties can negatively impact the performance of premixed calcium silicate-containing sealers, particularly AHB, when warm vertical compaction is employed.

## 1. Introduction

The aim of root canal obturation is to establish the hermetical seal of the cleaned and shaped root canal system and entomb any remaining microbial products with physically stable and biocompatible materials [1]. Root canal sealers are conventionally and commonly used together with gutta-percha and play an essential role in achieving the tight sealing of the root canal space [2]. However, root canal sealers are prone to exhibiting dissolution and shrinkage on a long-term basis [3]; thus, traditional root canal obturation techniques aim to achieve substantial gutta-percha filling with a minimal amount of sealer [4].

Warm vertical compaction aims to achieve the enhanced dentinal wall adaptation of gutta-percha by means of heat softening, facilitating filling into canal irregularities, particularly in the cervical third [5,6]. However, the heat produced during this procedure may deteriorate the physicochemical properties of some sealers; for example, heat application to epoxy-resin-based sealers results in a shorter curing time and an increase in film thickness owing to changes in bonding between benzene rings and aromatic amines [7,8]. Therefore, when employing warm vertical compaction, the choice of sealer should be considered for achieving tight and long-term sealing.

Recently, premixed calcium silicate-containing root canal sealers (CSCSs), which were developed by the modification of mineral trioxide aggregate (MTA), have been widely used. In common with the MTA, premixed CSCSs set via the hydration reaction of calcium silicates to form insoluble calcium silicate hydrate together with calcium hydroxide [9] and possess several favorable properties such as bioactivity, biocompatibility, sealing ability, and structural stability [10,11]. The modification includes the addition of water-free thickening vehicles to increase viscosity and confer handling properties suitable for root canal obturation [9]. The vehicles absorb environmental moisture, which is necessary for the hydration reaction and eventual setting. As in the MTA, premixed CSCSs exhibit bioactivity that primarily relies on the gradual release of calcium ions from calcium hydroxide and the subsequent surface precipitation of carbonated apatites; these reactions are responsible for the materials’ ability to induce biomineralization [12,13,14]. EndoSequence BC Sealer (a first-generation premixed CSCS; Brasseler, Savannah, GA, USA; ES) contains monobasic calcium phosphate, which reacts with calcium hydroxide and promotes the nucleation of carbonated apatites [15]. Premixed CSCSs are highly biocompatible [9,16,17] and capable of inducing favorable cellular responses, including the promotion of osteogenic gene expression and mineralized nodule formation in osteoblastic cells [16,17] and the suppression of proinflammatory cytokine expression in macrophages [17]. These findings provide a biological basis for the clinical application of these sealers.

A newly developed premixed CSCS, AH Plus Bioceramic Sealer (Dentsply Sirona, Charlotte, NC, USA; AHB), has recently been introduced. This product exclusively contains tri-calcium silicate [18,19] and has a lower calcium silicate percentage (5.0–15.0 wt% according to the Material Safety Data Sheet) compared to most other premixed CSCSs, such as ES, which contains 20.0–35.0 wt% of tri-calcium silicate and 7.0–15.0 wt% of di-calcium silicate according to the Material Safety Data Sheet. According to the manufacturer, AHB sets quickly, has a low washout rate, and is radiopaque.

A single-cone technique (hydraulic condensation technique) is recommended for root canal obturation with premixed CSCSs, owing to their high flowability, stability, and setting expansion [20]. However, the application of these sealers using warm vertical compaction remains controversial. Some studies have demonstrated that heating these sealers reduces the setting time and flow [8,21,22] and increases solubility [23], making proper filling challenging. In contrast, other studies have shown that heating has minimal impact on the sealers’ physicochemical properties [18,24,25]. Thus, whether and how heat application influences the physicochemical properties of various CSCSs warrants further investigation.

Regarding the clinical impact of heat application to premixed CSCSs, a few studies have reported favorable short-term outcomes [26,27,28,29]. In one study, a 1-year success rate of 99% was reported when four brands of premixed CSCSs were used with either the continuous wave of condensation technique or the carrier-based technique [27]. In another study, 82% of cases obturated with AHB using a carrier-based technique (Thermafil; Dentsply, Konstanz, Germany) exhibited complete healing after 1 year [28]. Comparable success rates have been reported for premixed CSCSs and an epoxy resin-based sealer (AH Plus, Dentsply Sirona) when used in warm vertical compaction using the SuperEndo System (B&L Biotech, Ansan, Republic of Korea) at 150 °C [26] or the Thermafil technique [29]. Further investigation is still warranted due to the paucity of well-controlled long-term studies.

Limited information is available on the changes in the physical properties of AHB caused by heating [18,19]. Therefore, this study aimed to examine how heating affects the setting time, flow, film thickness, and surface properties of AHB in comparison with ES and other root canal sealers. The null hypothesis aims to assert that heat application has no effect on AHB and other tested sealers in terms of the physical properties analyzed in this study.

## 2. Materials and Methods

Four materials were tested: two CSCSs, AHB and ES; AH Plus Jet (an epoxy-resin-based sealer, Dentsply Sirona; AH); and Pulp Canal Sealer (a zinc oxide–eugenol sealer, Kerr, Brea, CA, USA; PCS) (Table 1). PCS and AHP were mixed according to the instructions provided by the manufacturer. AHB and ES were injected from the syringe immediately before use. The setting time, flow, and film thickness were measured at different heating conditions, according to the ISO 6876:2012 standard [30]. Each experiment was conducted eight times.

### 2.1. Setting Time

AH and PCS were immediately filled into a stainless-steel mold (10 mm inner diameter and 2 mm height) immediately after mixing. AHB and ES require water for setting; thus, they were filled in a gypsum mold (10 mm inner diameter and 1 mm height) following ISO standards. The setting time was measured while incubating the specimens under different conditions, namely in a 37 °C thermostatic chamber (TVN480DA, Advantec, Tokyo, Japan) and in a 100 °C oven (007S, KDF, Kyoto, Japan) for 30 or 60 s, and then transferred to the 37 °C chamber. The molds for the 100 °C test were preheated to 100 °C in the oven. The setting time was determined according to ISO 6876; a Gilmore-type metric indenter (100 ± 0.5 g in weight and 2 ± 0.1 mm in tip diameter) was slowly dropped onto the surface of specimens at a 1 min interval until no indentation was observed.

### 2.2. Flow

Sealers (0.05 ± 0.005 mL) were dispensed on the center of a glass plate (40 × 40 mm, 5 mm thickness) with a syringe. After 180 ± 5 s, a glass plate of the same type was put on top, and a 100 g weight was applied to achieve a total weight of 120 ± 2 g. Ten minutes following the weight application, the diameters (maximum and minimum) of the compressed sealers were determined with ImageJ software (version 1.53k, National Institutes of Health, Bethesda, MD, USA). For each sealer, measurements were taken at room temperature (23 ± 2 °C) or after the sealer was kept in a 100 °C oven for 30 or 60 s. The glass plates for the tests performed at 100 °C were preheated to 100 °C in the oven. The flow test was repeated if there was a difference between the maximum and minimum diameters by ≥1 mm.

### 2.3. Film Thickness

The sealer was dispensed on the center of a 5 mm thick glass plate having a contact surface area of 200 ± 25 mm^2^ and overlaid with another glass plate of the same size. The specimens were kept at room temperature (23 ± 2 °C) or in a 100 °C oven for 30 or 60 s followed by room temperature. The glass plates were preheated to 100 °C before use in the 100 °C groups. At 180 ± 10 s after the sealer was placed, a 150 N vertical load was applied carefully to the top plate using a vertical motorized test stand (MX2-500N, Imada, Aichi, Japan). Ten minutes after the placement, the combined thickness of the glass plates and the sealer was determined using a micrometer (Digital Outside Micrometer, Niigata Seiki, Niigata, Japan). The difference in the thicknesses of the glass plates with and without the sealer was calculated and considered as the film thickness.

### 2.4. Scanning Electron Microscopy and Energy-Dispersive X-ray Spectroscopy

The sealers were filled into polytetrafluoroethylene molds (2 mm inner diameter, 5 mm height) and placed either in a 37 °C incubator or in an oven at 100 °C for 30 or 60 s, followed by being transferred to the incubator at 37 °C. Following storage for 48 h, the specimen’s surface was polished using SiC papers to #1200 and osmium-coated. The surface ultrastructure was examined using scanning electron microscopy (SEM; model JSM-7900F, JEOL, Tokyo, Japan), and elemental compositions were analyzed using energy-dispersive X-ray spectroscopy (EDS; model JED-2300, JEOL).

### 2.5. Statistics

Data were analyzed using SPSS software (version 28.0; IBM, Chicago, IL, USA). After the verification of data normality and variance homogeneity using the Shapiro–Wilk test and Levene test, respectively, one-way analysis of variance (ANOVA) and Tukey’s post hoc test were employed. The significance threshold was set at 0.05.

## 3. Results

Table 2 shows the results of setting time, flow, and film thickness for all the tested materials at different temperature conditions.

### 3.1. Setting Time

The recommended standard for setting time in ISO 6876:2012 is ≥30 min [30], and all sealers in this experiment satisfied this requirement at 37 °C. PCS recorded the longest setting time of 4063.1 ± 93.5 min, while the shortest was for ES (559.9 ± 12.2 min). After heat application at 100 °C for 30 or 60 s, all sealers demonstrated a significant setting time reduction (*p* < 0.05). Specifically, AHB had setting times of 13.0 ± 5.0 min and 2.4 ± 0.5 min after heating for 30 and 60 s, respectively, which were below the standard values. AH, ES, and PCS met the reference values.

### 3.2. Flow

The flow standard in ISO 6876:2012 is ≥17 mm [30]. All sealers satisfied this requirement at room temperature. However, after heating at 100 °C for 30 s and 60 s, the values of all sealers were significantly decreased compared to those at room temperature (*p* < 0.05). The decrease was particularly noticeable in AHB and ES. None of the heat-applied sealers, except for AH when heated for 30 s, satisfied the standard requirement.

### 3.3. Film Thickness

All the sealers satisfied the ISO 6876:2012 standard condition for film thickness (≤50 µm) [30] at room temperature. However, heating of AHB and ES at 100 °C for 30 s or 60 s caused a significant increase in film thickness values (*p* < 0.05) (exceeding the standard condition), with AHB being markedly high compared with the other sealers (*p* < 0.05).

### 3.4. Surface Ultrastructure and Elemental Composition

**Figure 1:**
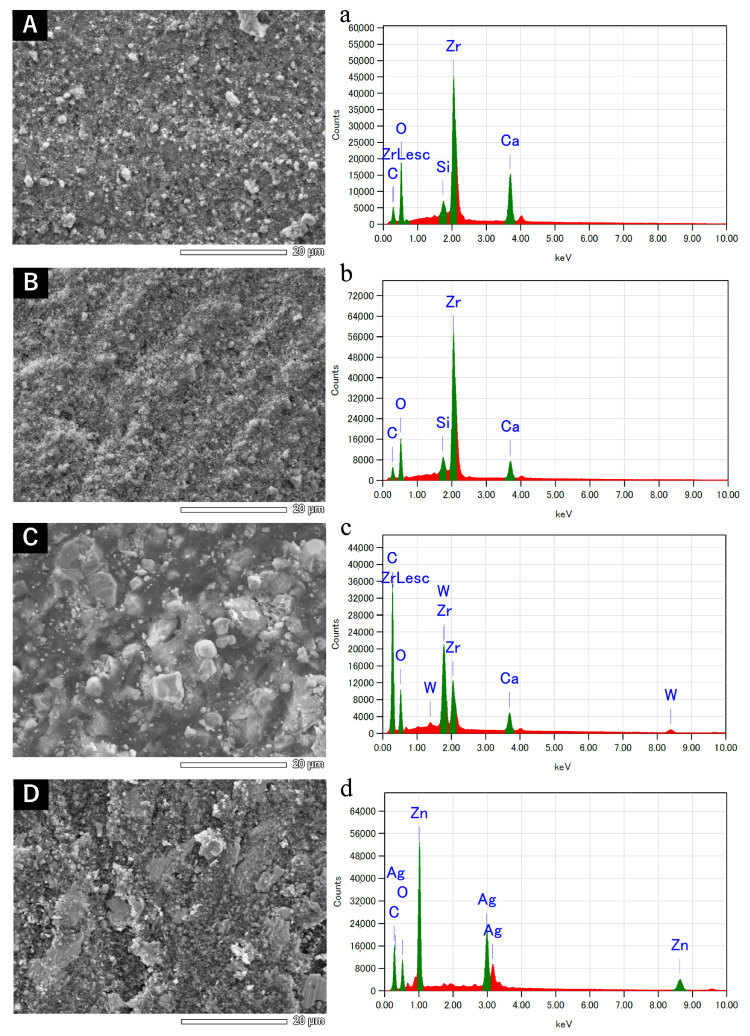
Backscatter scanning electron micrographs (**A**–**D**) and energy-dispersive X-ray microanalysis (**a**–**d**) of sealers left to set at 37 °C: (**A**,**a**) AH Plus Bioceramic Sealer, (**B**,**b**) EndoSequence BC Sealer, (**C**,**c**) AH Plus Jet, and (**D**,**d**) Pulp Canal Sealer.

**Figure 2:**
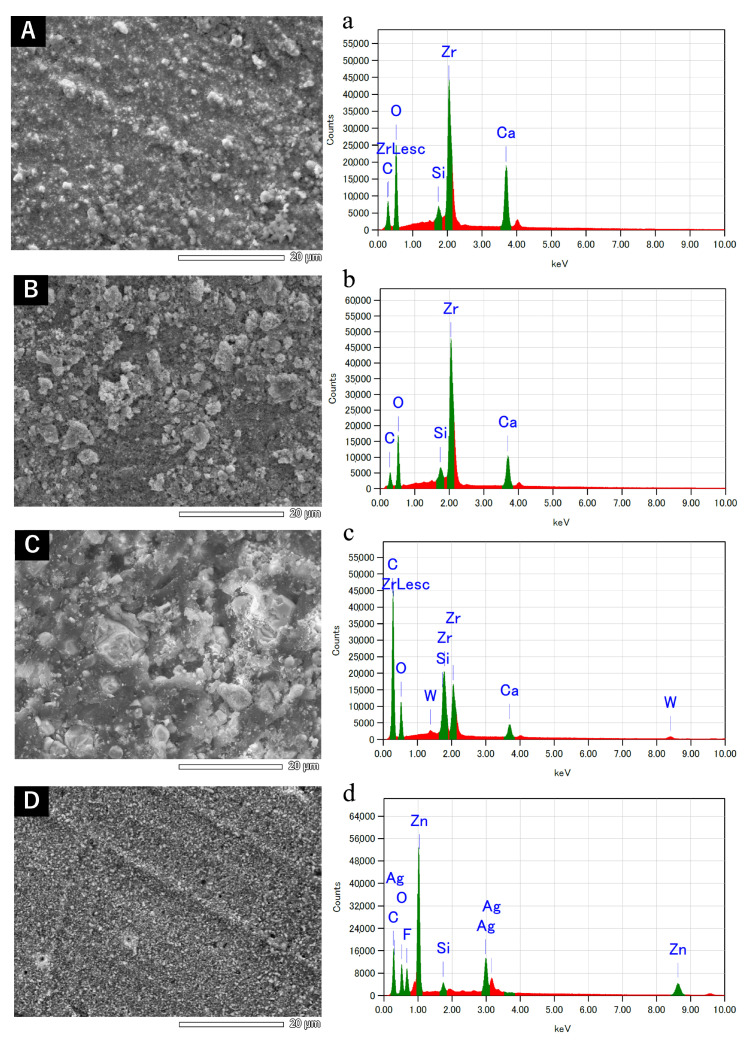
Backscatter scanning electron micrographs (**A**–**D**) and energy-dispersive X-ray microanalysis (**a**–**d**) of sealers subjected to heat application at 100 °C for 1 min and then left to set at 37 °C: (**A**,**a**) AH Plus Bioceramic Sealer, (**B**,**b**) EndoSequence BC Sealer, (**C**,**c**) AH Plus Jet, and (**D**,**d**) Pulp Canal Sealer.

Representative SEM images and EDS results are shown in Figure 1 and Figure 2.

No obvious changes in the surface properties were observed for AHB, ES, and AH when left to set at different temperature conditions. The SEM images of AHB showed white particles of approximately 1 µm in diameter. ES exhibited slightly larger particles (approximately 5 µm in diameter) and smaller particles (approximately 2 µm in diameter). The surface of AH showed relatively large white particles and a matrix. The SEM images of non-heated PCS featured particles approximately 5 µm in diameter. In heated PCS, the particle size decreased to approximately 0.6–1 µm.

EDS indicated the presence of zirconium, oxygen, calcium, carbon, and silicon in AHB and ES; carbon, oxygen, tungsten, zirconium, and calcium in AH; and zinc, carbon, silver, and sodium in PCS. No obvious differences in the elemental composition were observed between non-heat-applied and heat-applied sealers.

## 4. Discussion

In the present study, all the examined sealers showed significant reductions in the setting time and flow, along with a significant increase in film thickness in AHB and ES, when subjected to heat application at 100 °C for 30 s or 60 s. The null hypothesis was therefore rejected.

Although a temperature setting of approximately 200 °C is recommended for the heat plugger used for warm vertical compaction, previous research has reported that the actual temperature reaches approximately 100 °C [31,32]. Thus, to better simulate clinical conditions, a temperature of 100 °C was employed, with the duration of the heating being 30 s and 60 s. In addition, the glass plates and molds used were heated to 100 °C in advance to make the methods more uniform [8,21]. However, another study has reported that the root surface temperature hardly rises to 50 °C at 1 min after 3 s activation of heat pluggers [33], suggesting that the current temperature setting at 100 °C could be regarded as the extreme limit.

The setting time of a root canal sealer plays a critical role in endodontic procedures because it determines the time necessary for establishing the sealer’s appropriate seal and structural stability [34]. An extremely short setting time may compromise the sealer’s adaptation because of insufficient manipulation time allowed for the operator, leading to gaps and inadequate sealing. This study found that the setting time of AHB reduced significantly after heat application and did not conform to the ISO standard (>30 min). This may be attributed to the heat-induced acceleration of the hydration reaction of calcium silicate components [35,36]. Compared with ES, which consists of di-calcium silicate and tri-calcium silicate, AHB comprises only tri-calcium silicate, which has a faster setting reaction than di-calcium silicate [36]. Such differences in calcium silicate components may have some implications in the more pronounced heat-induced reduction in the setting time of AHB compared to ES. Lithium carbonate added to AHB is known to expedite the setting times of cement and concrete [37] and could also contribute to the shorter setting time of AHB. However, the lithium carbonate content is small (<0.5%, according to the Material Safety Data Sheet), and lithium could not be detected due to EDS specifications. The shortened setting time of heat-applied AH may result from the loss of amino groups and the accelerated polymerization of epoxy resins [8,34]. The setting time reduction in the heat-applied PCS may be attributed to the evaporation of the liquid component and the chemical disintegration of the component caused by heat [38]. However, some earlier studies have reported that the setting time was not affected by heating conditions of 100 °C for 60 s for the four sealers used in this study [18,38,39], which is in contrast to the present findings. Such a discrepancy may be attributed to the preheating of molds to 100 °C in this study, resulting in the effect of heat being greater than that in other studies.

Ensuring proper flow during root canal obturation is essential for allowing sealers to occupy irregularities and tightly seal the root canal space [1]. The flow of root canal sealers is influenced by various parameters, such as temperature, the particle size of the sealer, the inner diameter of the root canal, and the speed of insertion [1]. In this study, the flow of all the tested sealers met the ISO criteria (>17 mm) at room temperature, consistent with previous reports [40,41]. After heating at 100 °C for 30 and 60 s, however, the flow of all sealers significantly reduced and did not conform to the ISO standard. This reduction can be attributed to an accelerated setting reaction with increasing temperature [8]. Heat-applied AHB and ES showed a significantly lower flow than AH and PCS in most comparisons, which suggests the restricted compatibility of premixed CSCSs for use in warm vertical compaction.

Sealers are prone to shrinkage and dissolution over time [11,42]. Thus, it is believed that a thicker sealer layer negatively impacts the long-term maintenance of the root canal system’s hermetic sealing; consequently, using a minimal amount and a thin layer is recommended [4]. In the present study, AH and PCS met the ISO standard for film thickness (<50 µm) after heating at 100 °C for up to 60 s, whereas AHB and ES exceeded the standard after heating for up to 30 s and 60 s, respectively. Accelerated settings caused by heat may account for the increase in film thickness. However, CSCSs tend to expand by 0.2–6% [43], which is believed to be advantageous for achieving a tight seal [44] and could compensate for the negative impact of dissolution to some extent. In addition, the sealing ability is reported to vary depending on the sealer’s thickness and type [45]. Whether the thicker sealer layer created by CSCSs, particularly after heat application, maintains an acceptable long-term sealing quality warrants further investigation.

The SEM images of set ES, AHB, and AH revealed variations in particle size, elemental composition, and surface properties among the sealers, while no obvious changes caused by heating were discernible. Heat-applied PCS exhibited a decrease in particle size, which could be owing to the evaporation of eugenol upon heating, causing the reduced formation of zinc eugenolate chelate.

The reduced setting time and flow of heat-applied AHB could compromise the sealer’s adequate manipulation and spread during warm vertical compaction. ES showed a similar tendency, although its setting time was within the ISO standard. The heat-induced increase in the film thickness of AHB indicates that the advantages of warm vertical compaction, such as a thinner sealer layer owing to the higher gutta-percha-filled rate [5] and decreased porosity [46], may not be fully exploited.

The limitations of this study include the fact that the experiments were conducted at 100 °C, a temperature that could be different from actual clinical conditions, and the inability to evaluate the effects of changes in physical properties on the behavior of sealers in the root canal on a long-term basis. Application of chemical analysis with Raman spectroscopy and Fourier transform infrared spectroscopy [23] may increase our understanding of the modification of sealer components after heat application. Future studies are expected to explore long-term changes in the physical properties of sealers due to heating and the effects of their use in root canals on long-term prognosis.

## 5. Conclusions

In conclusion, under the present experimental condition, heat application resulted in a shortened setting time and reduced flow in all the sealers examined and caused an increased film thickness, most prominently in AHB, followed by ES. These alterations in physical properties could negatively impact the performance of premixed CSCSs, particularly AHB, when employed in warm vertical compaction.

## Figures and Tables

**Table 1:** Composition of materials used in this study.

Root Canal Sealer	Composition	Lot No.
AH Plus Bioceramic Sealer	Zirconium oxide, dimethyl sulfoxide, tricalcium silicate, lithium carbonate, thickening agents	KI210810
EndoSequence BC Sealer	Zirconium oxide, calcium silicates, calcium phosphate monobasic, calcium hydroxide, filler, thickening agents	19003SP
AH Plus Jet	Paste A: diepoxide, calcium tungstate, zirconium oxide, aerosol, pigment Paste B: 1-adamantane amine; N,N′-dibenzyl-5-oxa-nonandiamine-1,9; TCD diamine; calcium tungstate; zirconium oxide; aerosol silica; silicone oil	2202000427
Pulp Canal Sealer	Powder: zinc oxide, silver, resins, thymol iodide Liquid: eugenol, Canada balsams	9040827

**Table 2:** Setting time, flow, and film thickness at different temperature conditions.

Root Canal Sealer	Temperature Condition	Setting Time (min)	Flow (mm)	Film Thickness (µm)
AH Plus Bioceramic Sealer	37 °C/23 ± 2 °C ^§^	884.4 ± 40.5 ^Aa^	26.9 ± 2.5 ^Aa^	12.5 ± 2.4 ^Aa^
	100 °C 30 s ^#^	13.0 ± 5.0 ^Ba^	6.4 ± 0.6 ^Ba^	206.5 ± 59.4 ^Ba^
	100 °C 60 s ^##^	2.4 ± 0.5 ^Ca^	6.7 ± 0.6 ^Ba^	426.4 ± 109.6 ^Ca^
EndoSequence BC Sealer	37 °C/23 ± 2 °C	559.9 ± 12.2 ^Ab^	22.1 ± 1.0 ^Abc^	13.6 ± 1.8 ^Aa^
	100 °C 30 s	412.5 ± 13.1 ^Bb^	11.1 ± 2.5 ^Bb^	31.7 ± 6.0 ^Bbc^
	100 °C 60 s	233.3 ±1 6.3 ^Bb^	8.6 ± 1.0 ^Bab^	69.2 ± 9.5 ^Cb^
AH Plus Jet	37 °C/23 ± 2 °C	725.4 ± 34.6 ^Ac^	24.1 ± 0.9 ^Ab^	19.5 ± 2.5 ^Aa^
	100 °C 30 s	586.3 ± 12.5 ^Bc^	19.0 ± 2.2 ^Bc^	18.4 ± 4.1 ^Abc^
	100 °C 60 s	402.8 ± 45.0 ^Cc^	10.9 ± 1.0 ^Cbc^	21.1 ± 2.4 ^Ab^
Pulp Canal Sealer	37 °C/23 ± 2 °C	4063.1 ± 93.5 ^Ad^	21.6 ± 1.10 ^Ac^	16.0 ± 2.0 ^Aa^
	100 °C 30 s	2962.5 ± 16.3 ^Bd^	14.9 ± 0.8 ^Bd^	24.2 ± 2.5 ^Ab^
	100 °C 60 s	2558.8 ± 59.1 ^Cd^	11.7 ± 1.29 ^Cc^	24.9 ± 5.8 ^Ab^

^§^ Setting time was measured at 37 °C; flow and film thickness were measured at 23 ± 2 °C. ^#,##^ Sealers were subjected to heat application at 100 °C for 30 s ^#^ or 60 s ^##^, then left to set at 37 °C (setting time measurement) or 23 ± 2 °C (flow and film thickness measurements). The values represent the mean ± standard deviation of eight specimens. Values with different uppercase letters in the same column in each sealer, and values with different lowercase letters in each column in each temperature condition are significantly different at *p* < 0.05.

## Data Availability

The data presented in this study are available upon request from the corresponding author.
